# The Field of the A. V. M. A.

**Published:** 1902-05

**Authors:** 


### THE FIELD OF THE A. V. M. A.

The Journal wishes it could agree with its esteemed contemporary the *Review* on this all-important question. But the facts as they exist to-day cannot be ignored. The holding of surgical clinics finds its best place, its best work, in the field of local and State associations. It cannot be done under national auspices half so well, nor is it within their province when time is so limited to consider so many larger problems of general interest to our whole country. It is true that we should not ignore the general or routine practitioner, but if he is only interested in seeing some surgical clinics or hearing an every-day article on spavin, cunean tenotomy, or ovariotomy, we are at a loss to know what is added to truly National Association strength by his presence. If surgical clinics draw into the national organization a large number of local practitioners in every city where we hold our conventions, and whose faces are not seen again at our conventions for the next five years, or we again return to the city near his location, we cannot conceive how our association is to grow in national importance or power. If a cheap initiation fee and small dues are attracting a large membership numerically, who look upon it as a good investment, sub rosa an office-wall certificate, an annual report that serves as a year-book, and replaces entirely a subscription to one or more veterinary periodicals, then it will be well to keep on the present plan until every veterinarian in the land may have the opportunity by our presence in his vicinity to get in and avoid the rush.

Neither are we ready for sectional work in our association, for all efforts along this line so far have proven dismal failures, and lessened materially our progress and importance. Sectional work will come when we have gathered in a large majority of the foremost men in all the various lines of our work, and not until then. It will be time enough for this when we reach the point that men no longer decline membership or to give up so much valuable time attending A. V. M. A. conventions that devote half their time in the consideration of subjects that all well-posted older graduates should know, and of which all graduates of recent years eligible to association membership are thoroughly versed in. It is time that broader association work along truly national lines should attract the attention of a greater number of those who direct National and State work to our sessions, and not find them attending National Live-stock Associations, Breeders' organizations, State and National sanitary conventions, and National and State boards of health conventions to consider subjects that should first be presented and dwelt upon in the sessions of the A. V. M. A., and from there go to these other organizations as the refined judgment of the profession as a whole. No, we are not satisfied with the proposition to continue to travel along this rut any longer, nor to let this so-called well enough alone.

